# Spot the Difference? Contact Event Frequency During > 30,000 Women’s and Men’s Rugby Union Player Matches Across Top Domestic and International Competitions

**DOI:** 10.1002/ejsc.12307

**Published:** 2025-04-19

**Authors:** Gregory Roe, Tom Sawczuck, Neil Collins, James Tooby, Cameron Owen, Lindsay Starling, Éanna Falvey, Sharief Hendricks, Ross Tucker, Keith Stokes, Ben Jones

**Affiliations:** ^1^ Carnegie Applied Rugby Research (CARR) Centre Carnegie School of Sport Leeds Beckett University Leeds UK; ^2^ England Performance Unit Rugby Football League Manchester UK; ^3^ World Rugby Dublin Ireland; ^4^ School of Medicine & Health University College Cork Cork Ireland; ^5^ Division of Physiological Sciences and Health Through Physical Activity Lifestyle and Sport Research Centre Department of Human Biology Faculty of Health Sciences University of Cape Town Cape Town South Africa; ^6^ Institute of Sport and Exercise Medicine (ISEM) Department of Exercise University of Stellenbosch Stellenbosch South Africa; ^7^ Centre for Health and Injury and Illness Prevention in Sport University of Bath Bath UK; ^8^ UK Collaborating Centre on Injury and Illness Prevention in Sport (UKCCIIS) University of Bath Bath UK; ^9^ Rugby Football Union Twickenham UK; ^10^ Premiership Rugby London UK; ^11^ School of Behavioural and Health Sciences Faculty of Health Sciences Australian Catholic University Brisbane Australia

**Keywords:** collision sport, game demands, injury prevention, performance analysis

## Abstract

This study aimed to quantify the frequency of individual and team contact events during rugby union match play in top domestic and international men’s and women’s competitions. Analyst‐coded player individual and team contact event types (tackles, carries, attacking rucks and defensive rucks, lineouts, scrums and mauls) from the 2022/2023 rugby union season were analysed from top domestic and international competitions across the world using generalised linear mixed models. For both women’s and men’s rugby, competitions generally had similar numbers of contact events per playing position. Where differences were observed, most ranged between 0.5 and six per contact event per full game equivalent (FGE). Similar trends were observed when comparing women’s to men’s rugby. However, within‐game accumulation of these different contact events for certain positional groups may have a significant impact (e.g., a front five player called up from a Farah Palmer Cup team to play in WXV1 could be involved in as much as 6 more attacking rucks, 3 more tackles and 5 more mauls per game on average). Furthermore, the small differences between competitions per FGE may accrue across matches and thus result in far greater exposures across a season (e.g., a front five player in Premiership Rugby may make 48 more tackles over 20 matches than in Top 14 on average). Although a high proportion of contact events per FGE were similar between competitions and sexes per playing position, differences that were observed may have important implications for players transitioning between competitions and the long‐term exposure of players to higher‐risk contact events.

**Summary**

- In top domestic and international women’s and men’s rugby union competitions, players generally experienced similar numbers of contact events per playing position per FGE.
- However, differences observed in some positional groups may significantly affect the total number of contact events experienced per FGE when transitioning between domestic and international competitions and the exposure to certain contact events across a competitive season.
- Data presented provide a reference for practitioners working in top women’s and men's leagues across the world with which to guide conditioning of players for the demands of the game, whereas policymakers can use the information to inform decisions regarding player welfare, for example, strategies to reduce exposure to the most frequently occurring high‐risk contact events.

## 1 Introduction

Rugby union is a contact team sport played by both men and women globally. Contact events, such as the tackle, occur frequently during match play (Paul et al. 2022; Hendricks et al. 2018), thus success ratios (e.g., completed vs. missed tackles) and outcomes related to physical dominance (e.g., breaking an attempted tackle) are associated with team performance (Scott et al. 2023; Bennett et al. 2021). Within both women’s and men’s rugby union, the highest proportion of injuries also occur during tackles, ball‐carries into contact and rucks (Williams et al. 2022; Starling et al. 2023; West et al. 2022; Burger et al. 2020; King et al. 2019). Hence, contact events, particularly the tackle, have been a specific focus of injury mitigation research in recent years (Cross et al. 2019; Davidow et al. 2018; Meintjes et al. 2021).

Given the association of contact events with both performance and injury, the frequency of contacts that players may be involved in during match play is important information. Such information can be used in the physical preparation of players (Paul et al. 2022; Dane et al. 2022) both in single competitions and transitioning between competitions (e.g., from domestic to international; Beard et al. 2019; Tierney et al. 2021) and for designing position specific rehabilitation programmes (Villarejo et al. 2013; Sclafani and Davis 2016). Furthermore, from the perspective of governing bodies, understanding the contact events that players are exposed to during a match and at different levels of competition may help guide legislation, either individually, or in conjunction with other data sources. For example, combining contact event data with head acceleration event data can provide estimates of head acceleration exposure for different positions across matches, seasons and playing careers (Tooby et al. 2023). Such information may provide greater granularity with respect to a player’s risk of injury than current guidelines, which have been led by minutes played or match inclusions (Williams et al. 2023, 2017).

Despite the benefits of understanding contact event frequencies to a wide range of stakeholders, previous research in this area has mostly been restricted to low sample sizes, single teams or competitions with a bias towards male participants (Paul et al. 2022; Dane et al. 2022). Furthermore, the grouping of players into individual positional groups is inconsistent between studies limiting the comparisons that can be made (Paul et al. 2022; Dane et al. 2022). Thus, more research is required to understand players' exposure to contact events during matches, across competitions and sexes using standardised data collection and analysis methods. Using a single data source and evidence‐based positional groupings, the aim of the present study was to quantify and compare the frequency of individual and team contact events during rugby union match play in top domestic and international women’s and men’s competitions.

## 2 Materials and Methods

### 2.1 Study Design and Sample

A retrospective observational cohort study was conducted in rugby union players competing in top domestic and international competitions (i.e., established competitions at the highest level of domestic or international) across the world. Competitions were included if they were established (i.e., running for a number of years) and data were available from a commercial sports performance analysis company, StatsPerform (Chicago IL, United States). Competition and player information are provided in Table 1. Players were included whether they were a starter or a substitute. Accounting for the specific position played by players during each match, players were clustered into the following positional groups (Quarrie et al. 2013); front five, back row, halfbacks, outside back, and centres (Table 1). A total of 255,300 attacking rucks, 74,853 defensive rucks, 138,879 ball‐carries, 242,100 tackles, 38,366 lineouts, 21,990 scrums and 14,558 mauls were analysed across 32,038 player matches. Ethics approval was received from the university ethics committee (Ref: 123887).

**TABLE 1:** League and player information for the 22/23 domestic and international seasons.

		No. of players/player matches				
League	No. of matches/player matches	Front 5	Back row	Halfbacks	Centres	Outside backs
Men’s						
Domestic competitions						
Premiership rugby (professional)	119/5324	216/2072	107/994	76/771	63/633	95/854
Top 14 (professional)	187/8512	257/3418	124/1563	91/1214	80/1009	124/1308
United rugby championship (professional)	149/6740	303/2660	143/1240	108/1002	88/760	138/1078
Super rugby (professional)	91/4149	195/1618	84/751	66/604	61/508	78/668
Japan Rugby league one (professional)	91/4077	183/1531	91/779	71/590	58/471	93/706
International competitions						
Six nations	15/681	73/262	33/129	32/107	23/76	28/107
Rugby championship	12/545	55/205	25/110	24/84	14/57	22/89
World cup	48/2189	250/862	120/403	97/323	73/246	109/355
Women’s						
Domestic competitions						
Allianz premier 15s (semi‐professional)	88/3904	160/1496	79/728	57/578	46/420	79/682
Farah palmer cup (amateur)	42/1884	161/735	74/334	55/255	51/234	74/326
International competitions						
Six nations	15/674	71/254	33/128	24/99	25/87	29/106
WXV1	9/412	67/159	29/75	23/61	22/50	30/67
World cup	26/1162	136/455	68/220	52/165	43/139	57/183

### 2.2 Procedures

Match play contact event data were taken from Opta data (> 95% of all matches in each competition) using StatsPerform (Chicago IL, United States), a commercial sports performance analysis company which provides performance analysis data to numerous competitive sports leagues worldwide. Opta data were extracted online (https://www.optaprorugby.com/index.php) as Extensible Markup Language (XML) files. Contact events were coded by StatsPerform’s expert analysts at an individual level (individual contact events) including tackles, ball‐carries, attacking and defending rucks and at a team level (team contact events) including lineouts, scrums and mauls (Perform 2023). Opta data have demonstrated high interobserver reliability within football for team events, with kappa values of 0.92–0.94 (Liu et al. 2013). There has yet to be a similar investigation undertaken in rugby union, but data are used and trusted by professional clubs, broadcasters and other commercial organisations worldwide and are used in many studies in rugby union (Scott et al. 2023; Bennett et al. 2021, 2019).

### 2.3 Statistical Analysis

To estimate the frequency of contact events during rugby union match play, generalized linear mixed models were used. Two types of models were produced: one for individual contact events (attacking rucks, defensive rucks, carries and tackles) and one for team contact events (lineouts, mauls and scrums). Individual contact events were provided at the fixture level per player, with team contact events provided at the fixture level per team. This differentiation was necessary as the coding practices of Opta did not provide details of the individual players involved in these contact events. Within these types of models, two analyses were run: one considering differences between individual competitions and one considering differences between competition types (e.g., domestic/international) by sex. Consequently, four models were run in total.

For individual contact events, a negative binomial distribution was assumed. When comparing between individual competitions, contact event, positional group, competition and the logarithm of minutes played were used as fully factorial fixed effects. When comparing between types of competition and sex, contact event, positional group, sex, type of competition and the logarithm of minutes played were used as fully factorial fixed effects. The logarithm of minutes played was used in both models as it provided a better model fit than the raw minutes played and allowed contact event counts to be estimated per full game equivalent (FGE) whist accounting for the fact that individual players played different minutes. The random effect structure was consistent in both models, with random intercepts added for player ID (to account for repeated measurements within players) and fixture ID (to account for repeated measurements within fixtures).

For the team contact events, a Poisson distribution was assumed. When comparing between individual competitions, contact event and competition were included as fully factorial fixed effects. When comparing between types of competition and sex, contact event, competition type and sex were included as fully factorial fixed effects. As these analyses were conducted at the team level, there was no requirement for minutes played covariate to be included. In both models, fixture ID was included as a random effect.

Results are reported as least square means [95% confidence intervals] per FGE of match play (Williams et al. 2023; Sawczuk et al. 2024). Indicative differences were identified when the confidence intervals of the estimates did not overlap (Noguchi and Marmolejo‐Ramos 2016). Statistical analyses were completed in R (version 4.3.2) using the *glmmTMB* package (Brooks et al. 2017).

## 3 Results

**FIGURE 1:**
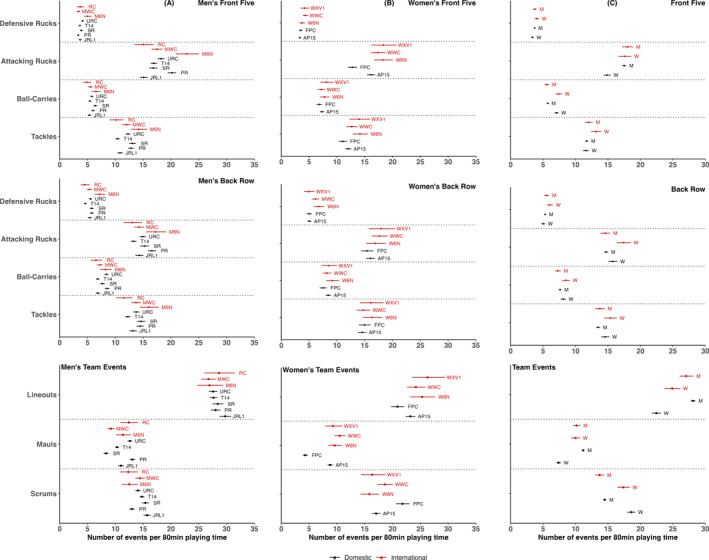
The per 80 min number of individual and team contact events broken down by competition for men’s forwards (A) and for women’s forwards (B) and the per 80 min number of individual and team contact events for forwards in domestic and international men’s and women’s competition (C). Data are mean and 95% confidence intervals. RC = rugby championship, MWC = men’s World Cup, M6N = men’s Six Nations, URC = United Rugby Championship, T14 = Top 14, SR = Super Rugby, PR = Premiership Rugby, JRL1 = Japan Rugby League 1. WXV1 = WXV1, WWC = women’s World Cup, W6N = women’s Six Nations, FPC = Farah Palmer Cup, AP15 = Allianz Premier 15s, W = women’s and M = men’s.

**FIGURE 2:**
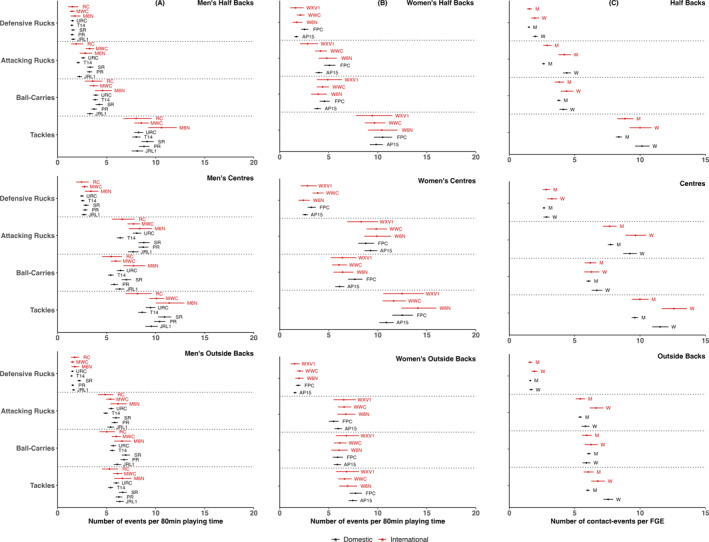
The per 80 min number of individual contact events broken down by competition for men’s backs (A) and for women’s backs (B) and the per 80 min number of individual contact events for backs in domestic and international men’s and women’s competition (C). Data are mean and 95% confidence intervals. RC = rugby championship, MWC = men’s World Cup, M6N = men’s Six Nations, URC = United Rugby Championship, T14 = Top 14, SR = Super Rugby, PR = Premiership Rugby, JRL1 = Japan Rugby League 1. WXV1 = WXV1, WWC = women’s World Cup, W6N = women’s Six Nations, FPC = Farah Palmer Cup, AP15 = Allianz Premier 15s, W = women’s and M = men’s.

Figure 1 shows the number of individual contact events, as well as team contact events per FGE, across all competitions for men’s and women’s forwards. Figure 2 presents the number of contact events for men’s and women’s backs per FGE across all competitions. Data are provided in numerical form in Tables S1–S6.

### 3.1 Forwards; Men’s Individual and Team Contact Events per Competition

In men (Figure 1A; Tables S1 and S3), front five players were involved in fewer defensive rucks in Top 14 (3.6 [3.4–3.8]) and Premiership Rugby (3.3 [3.1–3.5]) than in Men’s 6 Nations (5.0 [4.4–5.8]), fewer attacking rucks in all domestic European competitions compared to Men’s 6 Nations and fewer tackles in Top 14 (10.4 [10.0–10.8]) and URC (12.3 [11.8–12.7]) in comparison to Men’s 6 Nations (14.2 [12.8–15.7]). Back row experienced more defensive rucks in Men’s 6 Nations (7.2 [6.3–8.2]) than in the Top 14 (4.6 [4.4–4.8]), URC (5.5 [5.3–5.8)] and Premiership Rugby (5.8 [5.4–6.1]), greater numbers of attacking rucks in Men’s 6 Nations (17.2 [15.5–19.1]) than Top 14 (13.3 [12.7–13.8]), and a greater number of tackles in Men’s 6 nations (16.0 [14.4–17.8]) than in Top 14 (12.2 [11.7–12.7]) and URC (13.8 [13.2–14.3]). The number of lineouts was similar between domestic and international competitions. Super Rugby (8.3 [7.9–8.8]) had fewer mauls than the Rugby Championship (12.4 [10.9–14.1]), whereas there were fewer scrums in Premiership Rugby (13.0 [12.5–13.5]) than in the Men’s World Cup (14.4 [13.6–15.3]).

### 3.2 Forwards; Women’s Individual and Team Contact Events per Competition

In women (Figure 1B; Tables S2 and S4), front five players Farah Palmer Cup had significantly fewer attacking rucks (12.8 [12.0–13.6]) and tackles 11.0 [10.3–11.8]) than most other competitions, AP15s had lower tackles (12.0 [11.5–12.6] than Women’s 6 Nations (14.1 [12.8–15.6]), whereas international competitions were similar for all contact events. Back rows were involved in similar numbers of contact events in domestic and international competitions, except for defensive rucks, where the Women’s 6 Nations (6.7 [5.9–7.7]) were more frequent than in AP15s (5.1 [4.7–5.4]) and Farah Palmer Cup (5.0 [4.6–5.5]). For team events, Farah Palmer Cup had the lowest lineouts (20.9 [19.7–22.1]) and mauls (4.3 [3.9–4.8]) but the highest number of scrums (21.8 [20.6–23.0]) per FGE. Team events between AP15s and international competitions were not different except for mauls, of which AP15s were lower (8.8 [8.3–9.3]) than the Women's World Cup (10.5 [9.5–11.5]).

### 3.3 Forwards; Men’s versus Women’s Individual and Team Contact Events per Competition

When comparing men and women (Figure 1C; Tables S5 and S6), women’s front five were involved in more ball‐carries in domestic (7.1 [6.8–7.4] vs. 5.7 [5.7–6.0]) and international (7.4 [6.9–7.9] vs. 5.6 [5.2–5.9]) match play, whereas men’s front five were involved in more attacking rucks (17.5 [17.2–17.8] vs. 14.9 [14.3–15.4]) during domestic competition. Women’s players had greater ball‐carries (8.5 [7.9–9.1] vs. 7.3 [6.8–7.7]) and attacking rucks (17.4 [16.4–18.5] vs. 14.6 [13.9–15.4]) during international competition and greater tackles (14.6 [14.0–15.2] vs. 13.5 [13.2–13.8]) during domestic competition. Men’s domestic match play had more lineouts (28.1 [27.7–28.5] vs. 22.5 [21.8–23.2]) and mauls (11.2 [11.8–11.4] vs. 7.4 [7.0–7.7]), whereas women’s domestic (18.6 [18.0–19.2] vs. 14.5 [14.3–14.7]) and international (17.4 [16.4–18.4] vs. 13.7 [13.0–14.4]) competitions had a greater number of scrums.

### 3.4 Backs; Men’s Individual and Team Contact Events per Competition

For men (Figure 2A; Tables S1 and S3), during Men’s 6 Nations (10.6 [9.2–12.2]) halfbacks were involved in more tackles than in Top 14 (8.0 [7.6–8.5]) and United Rugby Championship (8.3 [7.8–8.7]). Similarly, centres were involved in fewer ball‐carries in the Top 14 (5.4 [5.1–5.7]) and Premiership Rugby (5.8 [5.4–6.2]) than the Men’s 6 Nations (7.7 [6.7–8.9]) and fewer tackles in Top 14 (8.6 [8.2–9.1]) than Men’s 6 Nations (11.4 [10.0–12.9]). Furthermore, centres in Men’s 6 Nations (11.4 [10.0–12.9]) performed a greater number of tackles than those in the Rugby Championship (8.1 [6.9–9.6]) and a greater number of ball‐carries (7.7 [6.7–8.9]) than in the Rugby Championship (5.5 [4.5–6.6]) and Men’s World Cup (5.9 [5.4–6.5]). In Super Rugby, centres performed more tackles (10.9 [10.2–11.6] vs. 8.1 [6.9–9.6]) and attacking rucks (8.8 [8.2–9.4] vs. 6.6 [5.5–7.8]) than in the Rugby Championship. Outside backs performed more ball‐carries in Super Rugby (6.9 [6.5–7.4]) than the Rugby Championship (5.0 [4.3–5.9]) and more defensive rucks in Super Rugby (2.2 [2.0–2.4]) than the World Cup (1.5 [1.3–1.7]).

### 3.5 Backs; Women’s Individual and Team Contact Events per Competition

For women (Figure 2B; Tables S2 and S4), halfbacks were involved in more attacking rucks (5.1 [4.5–5.7] vs. 4.0 [3.6–4.4]) and defensive rucks (2.5 [2.2–2.9] vs. 1.7 [1.5–1.9]) in Farah Palmer Cup compared to AP15s and more attacking rucks in Farah Palmer Cup than WXV1 (5.1 [4.5–5.7] versus. 2.8 [2.1–3.9]). Centres were involved in more ball‐carries in Farah Palmer Cup than AP15s (7.7 [7.0–8.4] vs. 6.1 [5.7–6.6]), lower defensive rucks in Farah Palmer Cup compared to the Women’s World Cup (2.6 [2.3–2.9] vs. 3.9 [3.3–4.5]) and lower tackles in Farah Palmer Cup compared to Women's 6 Nations (10.9 [10.2–11.6] vs. 14.1 [12.4–16.0]). Outside backs were involved in a similar number contact events in all competitions.

### 3.6 Backs; Men’s versus Women’s Individual and Team Contact Events per Competition

When comparing men and women (Figure 2C; Tables S5 and S6), women’s halfbacks were involved in more defensive rucks during domestic competition (2.0 [1.8–2.2] vs. 1.5 [1.4–1.6]), more attacking rucks in both domestic (4.4 [4.1–4.7] vs. 2.6 [2.5–2.7]) and international (4.2 [3.8–4.7] vs. 2.9 [2.6–3.2]) match play and more tackles during domestic matches (10.2 [9.6–10.7] vs. 8.4 [8.1–8.6]). For centres, women’s players were involved in more attacking rucks in both domestic (9.2 [8.7–9.8] vs. 7.8 [7.5–8.0]) and international (9.6 [8.9–10.5] vs. 7.7 [7.2–8.2]) competition and a greater number of tackles in match play at both domestic (11.5 [10.9–12.2] vs. 19.6 [9.4–9.9]) and international (12.6 [11.7–13.6] vs. 10.0 [9.4–10.6]) levels. In outside backs, women’s players were involved in greater attacking rucks during international competitions (6.6 [6.1–7.2] vs. 5.4 [5.1–5.8]) and greater tackles during domestic competition (7.6 [7.2–7.9] vs. 6.0 [5.9–6.2]).

## 4 Discussion

The aim of the present study was to describe the frequency of individual and team contact events during rugby union match play in top domestic and international women’s and men’s competitions using a single data source and evidence‐based positional groups. For both women’s and men’s rugby union, competitions generally had similar numbers of contact events per playing position and similar trends were observed when comparing women’s to men’s rugby union. Where differences were observed, most ranged between 0.5 and six per contact event type per FGE. However, the accumulation of contact events during a match for certain positional groups may have a significant impact. For example, a front five player called up from a Farah Palmer Cup team to play in WXV1 could be involved in up to 11 more contact events per FGE on average. Furthermore, the small differences between competitions per FGE may accrue across matches and thus result in higher exposures over the full duration of a competition. For example, a front five player in Premiership Rugby may make approximately 48 more tackles over 20 matches than in Top 14. Data presented in the present study provide a reference for practitioners working in top men’s and women’s leagues across the world on which to condition their players for the demands of the game, both in single competitions and transitioning in between. Furthermore, policymakers can use the information presented in this study to guide decisions regarding player welfare, for example, strategies to manage exposure to the most frequently occurring high‐risk contact‐events.

In the women’s competitions analysed, both forwards and backs had similar numbers of individual contact events per FGE during domestic match play in comparison to associated international competition for the most part (Figures 1 and 2). Where statistical differences were observed, absolute differences ranged from approximately 0.5 to 5 contact events per FGE. Similar trends were observed for international competitions and domestic competitions when considered separately. Nevertheless, recent research in the women’s game has demonstrated associations between collision outcome and physical characteristics such as body mass, strength and power (Woodhouse et al. 2023). Although no comparisons in physical characteristics have yet been made between playing levels in women’s rugby union (Curtis et al. 2023), data from women’s rugby league suggest that international players have greater anthropometric (height, body mass and fat‐free mass) and physical (strength, speed, power and aerobic capacity) qualities than domestic players (Scantlebury et al. 2022). Thus, it is possible that differences between domestic and international women’s rugby may be more pronounced with respect to contact event intensity than the number of contact events. However, future research is required to clarify this.

For team events in the women’s game, both Farah Palmer Cup (4.3 [3.9–4.8]) and AP15 (8.8 [8.3– 9.3]) had a lower number of mauls than the Women’s World Cup 10.5 (9.6–11.5), whereas Farah Palmer Cup also had less mauls than WXV1 (9.3 [7.9–11.0]) per match. Given that the maul produces a significant amount of neuromuscular and metabolic stress (Morel et al. 2015), forwards in these competitions may require additional position‐specific conditioning to optimally prepare for international match demands. Interestingly, Farah Palmer Cup had more scrums than any other women’s competition. As scrums primary result from mistakes in play (e.g., knock on), this difference may represent differences in the skill level (i.e., amateur vs. semi‐professional competitions).

Similar to the results in women’s rugby, overall results for men’s rugby demonstrated similarity between competitions with respect to the individual contact events that different playing positions were involved in per FGE (Figures 1 and 2). Although previous research exploring match demands in rugby union is typically hard to compare due to the plethora of methods used (Curtis et al. 2023), data from the same players competing in multiple competitions and playing levels suggest that match demands are greater at higher levels of competition (Tierney et al. 2021). In particular, the microtechnology‐derived accelerometer data that accumulates from collisions during match play have been shown to be highest in international competitions and greater in higher levels of club competitions (e.g., Pro14 vs. British and Irish Cup) (Tierney et al. 2021). Therefore, it is possible that although many positional groups were involved in a similar number of contact events across competitions, the intensity of these collisions may differ. Furthermore, the intensity or magnitude of collisions can be determined by the body load, or head accelerations event, measured using instrumented mouthguards (Jones et al. 2022).

Where differences among competitions were observed in men’s rugby union, the majority were between European domestic competitions and the Six Nations. Given that the Six Nations is played every year alongside domestic European competitions, it is important for physical preparation and rehabilitation practitioners to take stock of these differences when preparing players to transition between competitions (Beard et al. 2019; Tierney et al. 2021). This is particularly important for Top 14 players where the results demonstrated that front five had significantly fewer defensive rucks, tackles and attacking rucks; back row experienced fewer defensive rucks, attacking rucks and tackles; halfbacks were involved in less tackles and centres partook in less ball‐carries and tackles than in the Six Nations.

With respect to team contact events, the vast majority were similar between competitions. The notable exceptions were the number of mauls in Super Rugby (8.3 [7.9–8.8]) and scrums in Premiership Rugby (13.0 [12.5–13.5]), which were lower than in the Rugby Championship (12.3 [10.8–14.0]) and men’s World Cup (14.4 [13.6–15.3]), respectively, and all other domestic leagues.

For a large proportion of contact events and positional groups, no differences were observed between women’s and men’s rugby for an FGE at either domestic or international level. Where differences were present, women’s rugby players tended to have a higher (approximately 0.5–4) number of individual contact events per FGE of competition. For the forwards, these differences were mainly seen in ball carries and attacking rucks (Figure 1), whereas in backs, differences primarily occurred in tackle and attacking ruck events (Figure 2). The current findings are similar to the movement characteristics of match play, for which men’s and women’s are relatively equivocal (Jones et al. 2015; Bradley et al. 2020). Nevertheless, recent data from instrumented mouthguards have demonstrated the difference in head acceleration event magnitude between men’s and women’s forwards and backs during tackles, ball carries and rucks (Tooby et al. 2023; Roe et al. 2024). Contact events during men’s rugby had a greater propensity for resulting in a head acceleration event across all magnitudes investigated, suggesting that the collision intensity during the men’s game is significantly greater (e.g., between 2.82 and 3.32 times greater at higher magnitudes). Thus, although for certain events and positions, women’s players may be involved in slightly more contact events per match, the physical cost of contact is likely greater for men.

For team events, at a domestic level, there were more lineouts (28.1 [27.7–28.5] vs. 22.5 [21.8–23.2]) and mauls (11.1 [10.8–11.3] vs. 7.4 [7.0–7.7]) in the men’s game, which may reflect a preference to kick more. Conversely, there were more scrums in women’s rugby at both domestic (18.6 [18.0–19.2] vs. 14.5 [14.2–14.8]) and international (17.4 [16.4–18.4] vs. 13.7 [13.0–14.4]) levels, which may reflect a greater number of handling errors in the women’s game.

### 4.1 Limitations and Future Research

Both a strength and a limitation of the present study is the use of a single source of video analysis data commercially available from Opta (Stats Perform, London, United Kingdom). Currently, no reliability studies have been conducted on Opta data in rugby union, and a reliability assessment was not undertaken in the present study. However, Opta data have shown high reliability in football with kappa values of 0.92–0.94 (Liu et al. 2013) and are trusted by professional clubs, broadcasters and other commercial organisations worldwide and are used in many studies in rugby union (Scott et al. 2023; Bennett et al. 2021, 2019). Additionally, the analysis undertaken in the current study presented data per FGE only. As can be seen in Figures 1 and 2, even when confidence intervals overlapped, point estimates often differed. Thus, it is possible that these differences would have been magnified if multiple games were modelled (e.g., a half or full season). Therefore, future research is required to determine how contact events may accumulate across matches and competitions using longitudinal analysis techniques. Also, FGEs were used to allow for direct comparisons between competitions. Future studies may benefit from using other ways of normalising playing time, such as median playing time of starters. Moreover, only counts of contact events were considered within the present study. Measures of contact intensity are required to paint a full picture of the contact demands in and between competitions. Where differences were identified, further research is required to explore factors that may contribute to these differences (e.g., in‐game contextual factors such as skill level and discipline). Finally, as larger datasets with more leagues become available, and changes to competitions occur, such as increased professionalism and rules modifications, the analysis undertaken in the present study will require updating.

### 4.2 Practical Applications

The differences in contact events between competitions spanned a narrow range (approximately 0.5–six per contact event per FGE) for both women’s and men’s players. However, the summation of these differences across the different contact events within a match may have a significant effect on certain positional groups. For example, a front five player called up from a Farah Palmer Cup team to play in WXV1 could be involved in as much as 6 more attacking rucks, 3 more tackles and 5 more mauls per game on average (Figure 1). Similarly, a Top 14 back row player, who is selected for a Six Nations squad, on average may be exposed to up 3 more defensive rucks, 4 more attacking rucks, 4 more tackles and 1 extra maul per match (Figure 1). The physiological cost of these additional contact events within a single match may have a substantial impact on fatigue during competition (Morel et al. 2015) if players are not adequately prepared. Furthermore, such fatigue may compound across a competition and result in underperformance or predispose to injury. Accordingly, the data presented in this study may help guide practitioners working in top men’s and women’s leagues across the world in the physical preparation of players for the demands of the game, both in single competitions and transitioning in between.

Additionally, small differences between competitions may accrue across matches and thus result in far greater exposures across the full duration of a competition. For example, a front five player competing in Premiership Rugby may be expected to make more tackles on average per full match than in the Top 14 (Figure 1). Across 20 full game equivalents, this would equate to 48 more tackles. Given that the tackle is the contact event in which most injuries occur during rugby union match play (Williams et al. 2022), a front five player may be at greater risk of injury when playing in Premiership Rugby or in the initial period after joining a team in Premiership Rugby from Top 14. Accordingly, policymakers can use the information presented in this study to guide decisions regarding player welfare, for example, competition‐specific seasonal match limits (Williams et al. 2017) to reduce exposure to high‐risk contact events.

## 5 Conclusion

The aim of the present study was to describe the frequency of individual and team contact events during rugby union match play in top domestic and international men’s and women’s competitions using a single data source and evidence‐based positional groups. Although a high proportion of contact events per FGE were similar between competitions and sexes, differences that were observed may have important implications for players transitioning between competitions and the long‐term exposure of players to higher‐risk contact events. Data presented in this study provide a reference for practitioners working in top men’s and women’s leagues across the world with which to guide conditioning of players for the demands of the game, both in single competitions and transitioning in between. Furthermore, policymakers can use the information presented in this study to guide decisions regarding player welfare, for example, strategies to reduce exposure to the most frequently occurring high‐risk contact events.

## Ethics Statement

Ethics statement was received from Leeds Beckett University Ethics Committee (Ref: 123887).

## Conflicts of Interest

The authors declare no conflicts of interest.

## Supporting information

Supporting Information
