# Supplementary material for: Spot the Difference? Contact Event Frequency During > 30,000 Women’s and Men’s Rugby Union Player Matches Across Top Domestic and International Competitions
Source: Eur J Sport Sci. 2025 Apr 19;25(5):e12307. doi: 10.1002/ejsc.12307 (PMC12009008; doi:10.1002/ejsc.12307)
Supplement: Supplementary file 1 — Supporting Information [file EJSC-25-e12307-s001.docx]

**Supplementary Table 1:** The per FGE number of individual contact-events for each men’s positional group.

| **Position** | **Contact-event** | **Competition** | **Mean per FGE (CIs)** |
| --- | --- | --- | --- |
| Back Row | Attacking Ruck | Japan Rugby League 1 | 14.3 (13.6 to 15.1) |
|  |  | Men's 6 Nations | 17.2 (15.5 to 19.1) |
|  |  | Men's World Cup | 14.3 (13.4 to 15.2) |
|  |  | Premiership Rugby | 16.6 (15.9 to 17.3) |
|  |  | Rugby Championship | 13.0 (11.5 to 14.8) |
|  |  | Super Rugby | 15.2 (14.5 to 16.0) |
|  |  | Top 14 | 13.3 (12.7 to 13.8) |
|  |  | URC | 14.9 (14.3 to 15.5) |
|  | Ball-carry | Japan Rugby League 1 | 6.9 (6.5 to 7.3) |
|  |  | Men's 6 Nations | 8.3 (7.3 to 9.4) |
|  |  | Men's World Cup | 7.2 (6.7 to 7.8) |
|  |  | Premiership Rugby | 8.5 (8.1 to 9.0) |
|  |  | Rugby Championship | 6.5 (5.6 to 7.6) |
|  |  | Super Rugby | 7.7 (7.2 to 8.1) |
|  |  | Top 14 | 6.8 (6.5 to 7.2) |
|  |  | URC | 8.4 (8.0 to 8.8) |
|  | Defensive Ruck | Japan Rugby League 1 | 5.4 (5.1 to 5.8) |
|  |  | Men's 6 Nations | 7.2 (6.3 to 8.2) |
|  |  | Men's World Cup | 5.3 (4.9 to 5.8) |
|  |  | Premiership Rugby | 5.8 (5.4 to 6.1) |
|  |  | Rugby Championship | 4.5 (3.8 to 5.4) |
|  |  | Super Rugby | 5.7 (5.4 to 6.1) |
|  |  | Top 14 | 4.6 (4.4 to 4.8) |
|  |  | URC | 5.5 (5.3 to 5.8) |
|  | Tackle | Japan Rugby League 1 | 13.2 (12.5 to 13.9) |
|  |  | Men's 6 Nations | 16.0 (14.4 to 17.8) |
|  |  | Men's World Cup | 13.7 (12.9 to 14.6) |
|  |  | Premiership Rugby | 14.4 (13.8 to 15.1) |
|  |  | Rugby Championship | 11.5 (10.1 to 13.1) |
|  |  | Super Rugby | 14.6 (13.9 to 15.4) |
|  |  | Top 14 | 12.2 (11.7 to 12.7) |
|  |  | URC | 13.8 (13.2 to 14.3) |
| Centres | Attacking Ruck | Japan Rugby League 1 | 7.7 (7.2 to 8.3) |
|  |  | Men's 6 Nations | 8.4 (7.3 to 9.6) |
|  |  | Men's World Cup | 7.7 (7.1 to 8.4) |
|  |  | Premiership Rugby | 8.7 (8.2 to 9.3) |
|  |  | Rugby Championship | 6.6 (5.5 to 7.8) |
|  |  | Super Rugby | 8.8 (8.2 to 9.4) |
|  |  | Top 14 | 6.4 (6.1 to 6.7) |
|  |  | URC | 8.1 (7.7 to 8.5) |
|  | Ball-carry | Japan Rugby League 1 | 6.3 (5.9 to 6.8) |
|  |  | Men's 6 Nations | 7.7 (6.7 to 8.9) |
|  |  | Men's World Cup | 5.9 (5.4 to 6.5) |
|  |  | Premiership Rugby | 5.8 (5.4 to 6.2) |
|  |  | Rugby Championship | 5.5 (4.5 to 6.6) |
|  |  | Super Rugby | 7.0 (6.5 to 7.5) |
|  |  | Top 14 | 5.4 (5.1 to 5.7) |
|  |  | URC | 6.4 (6.0 to 6.8) |
|  | Defensive Ruck | Japan Rugby League 1 | 2.7 (2.4 to 3.0) |
|  |  | Men's 6 Nations | 3.4 (2.8 to 4.1) |
|  |  | Men's World Cup | 2.8 (2.4 to 3.1) |
|  |  | Premiership Rugby | 2.8 (2.6 to 3.0) |
|  |  | Rugby Championship | 2.5 (1.9 to 3.2) |
|  |  | Super Rugby | 2.9 (2.6 to 3.2) |
|  |  | Top 14 | 2.6 (2.4 to 2.7) |
|  |  | URC | 2.5 (2.3 to 2.7) |
|  | Tackle | Japan Rugby League 1 | 9.6 (8.9 to 10.2) |
|  |  | Men's 6 Nations | 11.4 (10.0 to 12.9) |
|  |  | Men's World Cup | 10.1 (9.3 to 10.9) |
|  |  | Premiership Rugby | 10.4 (9.8 to 11.0) |
|  |  | Rugby Championship | 8.1 (6.9 to 9.6) |
|  |  | Super Rugby | 10.9 (10.2 to 11.6) |
|  |  | Top 14 | 8.6 (8.2 to 9.1) |
|  |  | URC | 9.5 (9.0 to 10.0) |
| Front Five | Attacking Ruck | Japan Rugby League 1 | 15.1 (14.4 to 15.8) |
|  |  | Men's 6 Nations | 22.9 (20.9 to 25.1) |
|  |  | Men's World Cup | 17.5 (16.5 to 18.5) |
|  |  | Premiership Rugby | 20.2 (19.4 to 20.9) |
|  |  | Rugby Championship | 15.1 (13.5 to 16.8) |
|  |  | Super Rugby | 16.8 (16.1 to 17.6) |
|  |  | Top 14 | 16.9 (16.4 to 17.5) |
|  |  | URC | 18.2 (17.6 to 18.9) |
|  | Ball-carry | Japan Rugby League 1 | 5.4 (5.0 to 5.7) |
|  |  | Men's 6 Nations | 6.5 (5.8 to 7.4) |
|  |  | Men's World Cup | 5.5 (5.1 to 6.0) |
|  |  | Premiership Rugby | 6.0 (5.7 to 6.3) |
|  |  | Rugby Championship | 4.9 (4.2 to 5.7) |
|  |  | Super Rugby | 6.4 (6.0 to 6.7) |
|  |  | Top 14 | 5.4 (5.2 to 5.7) |
|  |  | URC | 5.8 (5.5 to 6.0) |
|  | Defensive Ruck | Japan Rugby League 1 | 3.7 (3.4 to 3.9) |
|  |  | Men's 6 Nations | 5.0 (4.4 to 5.8) |
|  |  | Men's World Cup | 3.4 (3.1 to 3.7) |
|  |  | Premiership Rugby | 3.3 (3.1 to 3.5) |
|  |  | Rugby Championship | 3.7 (3.1 to 4.4) |
|  |  | Super Rugby | 3.9 (3.6 to 4.1) |
|  |  | Top 14 | 3.6 (3.4 to 3.8) |
|  |  | URC | 4.1 (3.9 to 4.3) |
|  | Tackle | Japan Rugby League 1 | 10.8 (10.3 to 11.4) |
|  |  | Men's 6 Nations | 14.2 (12.8 to 15.7) |
|  |  | Men's World Cup | 12.0 (11.3 to 12.7) |
|  |  | Premiership Rugby | 12.8 (12.3 to 13.3) |
|  |  | Rugby Championship | 10.2 (9.0 to 11.5) |
|  |  | Super Rugby | 13.1 (12.5 to 13.7) |
|  |  | Top 14 | 10.4 (10.0 to 10.8) |
|  |  | URC | 12.3 (11.8 to 12.7) |
| Half Backs | Attacking Ruck | Japan Rugby League 1 | 2.2 (2.0 to 2.5) |
|  |  | Men's 6 Nations | 2.8 (2.3 to 3.5) |
|  |  | Men's World Cup | 3.3 (2.9 to 3.7) |
|  |  | Premiership Rugby | 3.2 (3.0 to 3.5) |
|  |  | Rugby Championship | 1.9 (1.4 to 2.6) |
|  |  | Super Rugby | 3.3 (3.0 to 3.7) |
|  |  | Top 14 | 2.1 (1.9 to 2.3) |
|  |  | URC | 2.6 (2.4 to 2.8) |
|  | Ball-carry | Japan Rugby League 1 | 3.3 (3.0 to 3.6) |
|  |  | Men's 6 Nations | 4.6 (3.8 to 5.5) |
|  |  | Men's World Cup | 3.7 (3.3 to 4.1) |
|  |  | Premiership Rugby | 3.7 (3.4 to 4.0) |
|  |  | Rugby Championship | 3.6 (2.8 to 4.6) |
|  |  | Super Rugby | 4.2 (3.9 to 4.6) |
|  |  | Top 14 | 3.8 (3.6 to 4.1) |
|  |  | URC | 3.9 (3.6 to 4.2) |
|  | Defensive Ruck | Japan Rugby League 1 | 1.6 (1.4 to 1.8) |
|  |  | Men's 6 Nations | 1.8 (1.3 to 2.3) |
|  |  | Men's World Cup | 1.5 (1.2 to 1.7) |
|  |  | Premiership Rugby | 1.5 (1.3 to 1.6) |
|  |  | Rugby Championship | 1.5 (1.1 to 2.1) |
|  |  | Super Rugby | 1.6 (1.4 to 1.8) |
|  |  | Top 14 | 1.5 (1.3 to 1.6) |
|  |  | URC | 1.5 (1.4 to 1.7) |
|  | Tackle | Japan Rugby League 1 | 8.1 (7.6 to 8.7) |
|  |  | Men's 6 Nations | 10.6 (9.2 to 12.2) |
|  |  | Men's World Cup | 8.5 (7.8 to 9.3) |
|  |  | Premiership Rugby | 8.8 (8.3 to 9.4) |
|  |  | Rugby Championship | 8.0 (6.7 to 9.6) |
|  |  | Super Rugby | 9.1 (8.5 to 9.8) |
|  |  | Top 14 | 8.0 (7.6 to 8.5) |
|  |  | URC | 8.3 (7.8 to 8.7) |
| Outside Backs | Attacking Ruck | Japan Rugby League 1 | 5.4 (5.1 to 5.8) |
|  |  | Men's 6 Nations | 6.2 (5.4 to 7.0) |
|  |  | Men's World Cup | 5.4 (5.0 to 5.8) |
|  |  | Premiership Rugby | 5.8 (5.5 to 6.2) |
|  |  | Rugby Championship | 4.8 (4.1 to 5.7) |
|  |  | Super Rugby | 6.0 (5.6 to 6.3) |
|  |  | Top 14 | 4.9 (4.7 to 5.1) |
|  |  | URC | 5.5 (5.2 to 5.7) |
|  | Ball-carry | Japan Rugby League 1 | 6.1 (5.7 to 6.5) |
|  |  | Men's 6 Nations | 6.6 (5.8 to 7.5) |
|  |  | Men's World Cup | 6.0 (5.5 to 6.5) |
|  |  | Premiership Rugby | 6.8 (6.4 to 7.2) |
|  |  | Rugby Championship | 5.0 (4.3 to 5.9) |
|  |  | Super Rugby | 6.9 (6.5 to 7.4) |
|  |  | Top 14 | 5.6 (5.3 to 5.8) |
|  |  | URC | 5.7 (5.4 to 6.0) |
|  | Defensive Ruck | Japan Rugby League 1 | 1.6 (1.5 to 1.8) |
|  |  | Men's 6 Nations | 1.8 (1.4 to 2.2) |
|  |  | Men's World Cup | 1.5 (1.3 to 1.7) |
|  |  | Premiership Rugby | 1.5 (1.4 to 1.7) |
|  |  | Rugby Championship | 1.7 (1.4 to 2.2) |
|  |  | Super Rugby | 2.2 (2.0 to 2.4) |
|  |  | Top 14 | 1.4 (1.3 to 1.5) |
|  |  | URC | 1.5 (1.4 to 1.6) |
|  | Tackle | Japan Rugby League 1 | 6.3 (6.0 to 6.7) |
|  |  | Men's 6 Nations | 6.6 (5.8 to 7.5) |
|  |  | Men's World Cup | 6.1 (5.7 to 6.6) |
|  |  | Premiership Rugby | 6.2 (5.9 to 6.6) |
|  |  | Rugby Championship | 5.3 (4.5 to 6.2) |
|  |  | Super Rugby | 6.6 (6.2 to 7.1) |
|  |  | Top 14 | 5.4 (5.2 to 5.7) |
|  |  | URC | 6.0 (5.7 to 6.3) |

**Supplementary Table 2:** The per FGE number of individual contact-events for each women’s positional group.

| **Position** | **Contact-event** | **Competition** | **Mean per FGE  (CIs)** |
| --- | --- | --- | --- |
| Back Row | Attacking Rucks | AP15s | 16.0 (15.2 to 16.9) |
|  |  | Farah Palmer | 15.4 (14.3 to 16.5) |
|  |  | Women's 6 Nations | 16.9 (15.2 to 18.8) |
|  |  | Women's World Cup | 17.7 (16.3 to 19.1) |
|  |  | WXV | 17.9 (15.8 to 20.4) |
|  | Ball-carries | AP15s | 8.4 (8.0 to 8.9) |
|  |  | Farah Palmer | 7.5 (6.9 to 8.2) |
|  |  | Women's 6 Nations | 9.1 (8.1 to 10.3) |
|  |  | Women's World Cup | 8.2 (7.4 to 9.0) |
|  |  | WXV | 8.5 (7.3 to 10.0) |
|  | Defensive Rucks | AP15s | 5.1 (4.7 to 5.4) |
|  |  | Farah Palmer | 5.0 (4.6 to 5.5) |
|  |  | Women's 6 Nations | 6.7 (5.9 to 7.7) |
|  |  | Women's World Cup | 6.1 (5.5 to 6.8) |
|  |  | WXV | 4.9 (4.1 to 6.0) |
|  | Tackles | AP15s | 14.5 (13.8 to 15.3) |
|  |  | Farah Palmer | 15.0 (13.9 to 16.1) |
|  |  | Women's 6 Nations | 16.3 (14.7 to 18.1) |
|  |  | Women's World Cup | 14.7 (13.6 to 16.0) |
|  |  | WXV | 16.1 (14.1 to 18.4) |
| Centres | Attacking Rucks | AP15s | 9.3 (8.6 to 9.9) |
|  |  | Farah Palmer | 8.8 (8.0 to 9.6) |
|  |  | Women's 6 Nations | 9.9 (8.6 to 11.4) |
|  |  | Women's World Cup | 9.8 (8.8 to 10.9) |
|  |  | WXV | 8.3 (6.9 to 10.0) |
|  | Ball-carries | AP15s | 6.1 (5.7 to 6.6) |
|  |  | Farah Palmer | 7.7 (7.0 to 8.4) |
|  |  | Women's 6 Nations | 6.4 (5.5 to 7.5) |
|  |  | Women's World Cup | 6.0 (5.3 to 6.8) |
|  |  | WXV | 6.4 (5.2 to 7.8) |
|  | Defensive Rucks | AP15s | 2.6 (2.3 to 2.9) |
|  |  | Farah Palmer | 3.2 (2.8 to 3.7) |
|  |  | Women's 6 Nations | 2.4 (1.9 to 3.1) |
|  |  | Women's World Cup | 3.9 (3.3 to 4.5) |
|  |  | WXV | 2.8 (2.1 to 3.8) |
|  | Tackles | AP15s | 10.9 (10.2 to 11.6) |
|  |  | Farah Palmer | 12.5 (11.5 to 13.6) |
|  |  | Women's 6 Nations | 14.1 (12.4 to 16.0) |
|  |  | Women's World Cup | 11.6 (10.5 to 12.9) |
|  |  | WXV | 12.5 (10.6 to 14.7) |
| Front Five | Attacking Rucks | AP15s | 16.1 (15.4 to 16.9) |
|  |  | Farah Palmer | 12.8 (12.0 to 13.6) |
|  |  | Women's 6 Nations | 18.3 (16.7 to 20.1) |
|  |  | Women's World Cup | 17.4 (16.1 to 18.7) |
|  |  | WXV | 18.4 (16.2 to 20.8) |
|  | Ball-carries | AP15s | 7.3 (6.9 to 7.7) |
|  |  | Farah Palmer | 6.8 (6.3 to 7.3) |
|  |  | Women's 6 Nations | 7.8 (6.9 to 8.7) |
|  |  | Women's World Cup | 7.2 (6.5 to 7.9) |
|  |  | WXV | 8.1 (7.0 to 9.4) |
|  | Defensive Rucks | AP15s | 3.3 (3.1 to 3.6) |
|  |  | Farah Palmer | 3.5 (3.2 to 3.8) |
|  |  | Women's 6 Nations | 3.7 (3.2 to 4.3) |
|  |  | Women's World Cup | 4.3 (3.9 to 4.8) |
|  |  | WXV | 4.2 (3.4 to 5.1) |
|  | Tackles | AP15s | 12.0 (11.5 to 12.6) |
|  |  | Farah Palmer | 11.0 (10.3 to 11.8) |
|  |  | Women's 6 Nations | 14.1 (12.8 to 15.6) |
|  |  | Women's World Cup | 12.6 (11.7 to 13.7) |
|  |  | WXV | 14.0 (12.3 to 16.0) |
| Half Backs | Attacking Rucks | AP15s | 4.0 (3.6 to 4.4) |
|  |  | Farah Palmer | 5.1 (4.5 to 5.7) |
|  |  | Women's 6 Nations | 4.8 (4.0 to 5.8) |
|  |  | Women's World Cup | 4.2 (3.6 to 4.8) |
|  |  | WXV | 2.8 (2.1 to 3.9) |
|  | Ball-carries | AP15s | 3.8 (3.5 to 4.2) |
|  |  | Farah Palmer | 4.6 (4.1 to 5.1) |
|  |  | Women's 6 Nations | 3.9 (3.2 to 4.8) |
|  |  | Women's World Cup | 4.4 (3.8 to 5.0) |
|  |  | WXV | 4.9 (3.8 to 6.3) |
|  | Defensive Rucks | AP15s | 1.7 (1.5 to 1.9) |
|  |  | Farah Palmer | 2.5 (2.2 to 2.9) |
|  |  | Women's 6 Nations | 1.7 (1.3 to 2.3) |
|  |  | Women's World Cup | 2.1 (1.7 to 2.5) |
|  |  | WXV | 1.6 (1.1 to 2.4) |
|  | Tackles | AP15s | 9.8 (9.2 to 10.5) |
|  |  | Farah Palmer | 10.5 (9.6 to 11.5) |
|  |  | Women's 6 Nations | 10.4 (9.0 to 12.0) |
|  |  | Women's World Cup | 9.7 (8.7 to 10.8) |
|  |  | WXV | 9.4 (7.8 to 11.5) |
| Outside Backs | Attacking Rucks | AP15s | 6.0 (5.6 to 6.3) |
|  |  | Farah Palmer | 5.5 (5.0 to 6.0) |
|  |  | Women's 6 Nations | 6.7 (5.9 to 7.7) |
|  |  | Women's World Cup | 6.6 (5.9 to 7.3) |
|  |  | WXV | 6.5 (5.5 to 7.8) |
|  | Ball-carries | AP15s | 5.9 (5.5 to 6.3) |
|  |  | Farah Palmer | 5.9 (5.4 to 6.4) |
|  |  | Women's 6 Nations | 6.0 (5.3 to 6.9) |
|  |  | Women's World Cup | 6.1 (5.5 to 6.8) |
|  |  | WXV | 6.8 (5.7 to 8.1) |
|  | Defensive Rucks | AP15s | 1.6 (1.4 to 1.7) |
|  |  | Farah Palmer | 1.9 (1.6 to 2.1) |
|  |  | Women's 6 Nations | 2.0 (1.6 to 2.4) |
|  |  | Women's World Cup | 2.0 (1.7 to 2.4) |
|  |  | WXV | 1.5 (1.1 to 2.0) |
|  | Tackles | AP15s | 7.4 (7.0 to 7.9) |
|  |  | Farah Palmer | 7.7 (7.1 to 8.4) |
|  |  | Women's 6 Nations | 6.9 (6.1 to 7.9) |
|  |  | Women's World Cup | 6.6 (6.0 to 7.3) |
|  |  | WXV | 6.8 (5.8 to 8.1) |

**Supplementary Table 3:** The per FGE number of team contact-events for men’s rugby competitions.

| **Event** | **Competition** | **Mean per FGE (CIs)** |
| --- | --- | --- |
| Scrums | Japan Rugby League 1 | 15.7 (15.1 to 16.4) |
|  | Men's 6 Nations | 12.5 (11.2 to 14.0) |
|  | Men's World Cup | 14.4 (13.6 to 15.3) |
|  | Premiership Rugby | 13.0 (12.5 to 13.5) |
|  | Rugby Championship | 12.3 (10.8 to 14.0) |
|  | Super Rugby | 15.4 (14.8 to 16.1) |
|  | Top 14 | 14.8 (14.3 to 15.2) |
|  | URC | 14.1 (13.6 to 14.6) |
| Mauls | Japan Rugby League 1 | 11.0 (10.5 to 11.6) |
|  | Men's 6 Nations | 11.4 (10.1 to 12.8) |
|  | Men's World Cup | 9.2 (8.6 to 9.9) |
|  | Premiership Rugby | 13.1 (12.5 to 13.6) |
|  | Rugby Championship | 12.4 (10.9 to 14.1) |
|  | Super Rugby | 8.3 (7.9 to 8.8) |
|  | Top 14 | 10.3 (10.0 to 10.7) |
|  | URC | 12.6 (12.2 to 13.1) |
| Lineouts | Japan Rugby League 1 | 29.8 (28.8 to 30.8) |
|  | Men's 6 Nations | 26.9 (24.7 to 29.4) |
|  | Men's World Cup | 26.7 (25.5 to 28.1) |
|  | Premiership Rugby | 28.0 (27.2 to 28.9) |
|  | Rugby Championship | 28.6 (26.0 to 31.5) |
|  | Super Rugby | 28.4 (27.5 to 29.4) |
|  | Top 14 | 27.7 (27.0 to 28.3) |
|  | URC | 27.6 (26.8 to 28.3) |

**Supplementary Table 4:** The per FGE number of team contact-events for women’s rugby competitions.

| **Event** | **Competition** | **Mean per FGE (CIs)** |
| --- | --- | --- |
| Scrums | AP15s | 17.1 (16.4 to 17.8) |
|  | Farah Palmer | 21.8 (20.6 to 23.0) |
|  | Women's 6 Nations | 15.8 (14.3 to 17.6) |
|  | Women's World Cup | 18.6 (17.3 to 20.1) |
|  | WXV | 16.4 (14.3 to 18.7) |
| Mauls | AP15s | 8.8 (8.3 to 9.3) |
|  | Farah Palmer | 4.3 (3.9 to 4.8) |
|  | Women's 6 Nations | 9.6 (8.5 to 10.9) |
|  | Women's World Cup | 10.5 (9.5 to 11.5) |
|  | WXV | 9.3 (7.9 to 11.0) |
| Lineouts | AP15s | 23.2 (22.4 to 24.1) |
|  | Farah Palmer | 20.9 (19.7 to 22.1) |
|  | Women's 6 Nations | 25.3 (23.2 to 27.7) |
|  | Women's World Cup | 24.2 (22.6 to 25.9) |
|  | WXV | 26.3 (23.5 to 29.5) |

**Supplementary Table 5:** The per FGE number of individual contact-events for each men’s and women’s positional groups overall.

| **Position** | **Event** | **Sex** | **Type** | **Mean per FGE (CIs)** |
| --- | --- | --- | --- | --- |
| Back Row | Attacking Rucks | Women's | Domestic | 15.8 (15.1 to 16.4) |
|  |  |  | International | 17.4 (16.4 to 18.5) |
|  |  | Men's | Domestic | 14.7 (14.4 to 15.0) |
|  |  |  | International | 14.6 (13.9 to 15.4) |
|  | Ball-carries | Women's | Domestic | 8.1 (7.7 to 8.5) |
|  |  |  | International | 8.5 (7.9 to 9.1) |
|  |  | Men's | Domestic | 7.6 (7.4 to 7.8) |
|  |  |  | International | 7.3 (6.8 to 7.7) |
|  | Defensive Rucks | Women's | Domestic | 5.0 (4.7 to 5.3) |
|  |  |  | International | 6.0 (5.6 to 6.5) |
|  |  | Men's | Domestic | 5.3 (5.2 to 5.5) |
|  |  |  | International | 5.5 (5.2 to 5.9) |
|  | Tackles | Women's | Domestic | 14.6 (14.0 to 15.2) |
|  |  |  | International | 15.4 (14.4 to 16.4) |
|  |  | Men's | Domestic | 13.5 (13.2 to 13.8) |
|  |  |  | International | 13.8 (13.1 to 14.5) |
| Centres | Attacking Rucks | Women's | Domestic | 9.2 (8.7 to 9.8) |
|  |  |  | International | 9.6 (8.9 to 10.5) |
|  |  | Men's | Domestic | 7.8 (7.5 to 8.0) |
|  |  |  | International | 7.7 (7.2 to 8.2) |
|  | Ball-carries | Women's | Domestic | 6.7 (6.3 to 7.1) |
|  |  |  | International | 6.3 (5.7 to 6.9) |
|  |  | Men's | Domestic | 6.1 (5.9 to 6.2) |
|  |  |  | International | 6.2 (5.8 to 6.7) |
|  | Defensive Rucks | Women's | Domestic | 2.8 (2.6 to 3.1) |
|  |  |  | International | 3.3 (2.9 to 3.7) |
|  |  | Men's | Domestic | 2.7 (2.6 to 2.8) |
|  |  |  | International | 2.8 (2.6 to 3.1) |
|  | Tackles | Women's | Domestic | 11.5 (10.9 to 12.2) |
|  |  |  | International | 12.6 (11.7 to 13.6) |
|  |  | Men's | Domestic | 9.6 (9.4 to 9.9) |
|  |  |  | International | 10.0 (9.4 to 10.6) |
| Front Five | Attacking Rucks | Women's | Domestic | 14.9 (14.3 to 15.4) |
|  |  |  | International | 17.6 (16.6 to 18.6) |
|  |  | Men's | Domestic | 17.5 (17.2 to 17.8) |
|  |  |  | International | 18.0 (17.2 to 18.9) |
|  | Ball-carries | Women's | Domestic | 7.1 (6.8 to 7.4) |
|  |  |  | International | 7.4 (6.9 to 7.9) |
|  |  | Men's | Domestic | 5.7 (5.6 to 5.8) |
|  |  |  | International | 5.6 (5.2 to 5.9) |
|  | Defensive Rucks | Women's | Domestic | 3.4 (3.2 to 3.5) |
|  |  |  | International | 4.0 (3.7 to 4.4) |
|  |  | Men's | Domestic | 3.7 (3.6 to 3.8) |
|  |  |  | International | 3.7 (3.5 to 4.0) |
|  | Tackles | Women's | Domestic | 11.6 (11.1 to 12.1) |
|  |  |  | International | 13.2 (12.4 to 14.0) |
|  |  | Men's | Domestic | 11.7 (11.5 to 11.9) |
|  |  |  | International | 12.0 (11.4 to 12.6) |
| Half Backs | Attacking Rucks | Women's | Domestic | 4.4 (4.1 to 4.7) |
|  |  |  | International | 4.2 (3.8 to 4.7) |
|  |  | Men's | Domestic | 2.6 (2.5 to 2.7) |
|  |  |  | International | 2.9 (2.6 to 3.2) |
|  | Ball-carries | Women's | Domestic | 4.1 (3.8 to 4.4) |
|  |  |  | International | 4.4 (3.9 to 4.9) |
|  |  | Men's | Domestic | 3.8 (3.7 to 3.9) |
|  |  |  | International | 3.8 (3.5 to 4.2) |
|  | Defensive Rucks | Women's | Domestic | 2.0 (1.8 to 2.2) |
|  |  |  | International | 2.0 (1.7 to 2.3) |
|  |  | Men's | Domestic | 1.5 (1.4 to 1.6) |
|  |  |  | International | 1.5 (1.3 to 1.8) |
|  | Tackles | Women's | Domestic | 10.2 (9.6 to 10.7) |
|  |  |  | International | 10.0 (9.2 to 10.9) |
|  |  | Men's | Domestic | 8.4 (8.1 to 8.6) |
|  |  |  | International | 8.8 (8.3 to 9.5) |
| Outside Backs | Attacking Rucks | Women's | Domestic | 5.8 (5.5 to 6.1) |
|  |  |  | International | 6.6 (6.1 to 7.2) |
|  |  | Men's | Domestic | 5.4 (5.3 to 5.6) |
|  |  |  | International | 5.4 (5.1 to 5.8) |
|  | Ball-carries | Women's | Domestic | 5.9 (5.6 to 6.2) |
|  |  |  | International | 6.2 (5.8 to 6.8) |
|  |  | Men's | Domestic | 6.1 (5.9 to 6.2) |
|  |  |  | International | 5.9 (5.6 to 6.3) |
|  | Defensive Rucks | Women's | Domestic | 1.7 (1.5 to 1.8) |
|  |  |  | International | 1.9 (1.7 to 2.2) |
|  |  | Men's | Domestic | 1.6 (1.5 to 1.7) |
|  |  |  | International | 1.6 (1.4 to 1.8) |
|  | Tackles | Women's | Domestic | 7.6 (7.2 to 7.9) |
|  |  |  | International | 6.8 (6.3 to 7.3) |
|  |  | Men's | Domestic | 6.0 (5.9 to 6.2) |
|  |  |  | International | 6.0 (5.7 to 6.4) |

**Supplementary Table 6:** The per FGE number of team contact-events for men’s and women’s players overall.

| **Event** | **Sex** | **Type** | **Mean per FGE (CIs)** |
| --- | --- | --- | --- |
| Lineouts | Women's | Domestic | 22.5 (21.8 to 23.2) |
|  |  | International | 24.9 (23.7 to 26.2) |
|  | Men's | Domestic | 28.1 (27.7 to 28.5) |
|  |  | International | 27.1 (26.0 to 28.2) |
| Mauls | Women's | Domestic | 7.4 (7.0 to 7.7) |
|  |  | International | 10.0 (9.3 to 10.7) |
|  | Men's | Domestic | 11.2 (11.0 to 11.4) |
|  |  | International | 10.2 (9.6 to 10.7) |
| Scrums | Women's | Domestic | 18.6 (18.0 to 19.2) |
|  |  | International | 17.4 (16.4 to 18.4) |
|  | Men's | Domestic | 14.5 (14.3 to 14.7) |
|  |  | International | 13.7 (13.0 to 14.4) |
